# The complete chloroplast genome of *Lonicera hypoglauca* Miq (Caprifoliaceae: Dipsacales) from Guangxi, China

**DOI:** 10.1080/23802359.2020.1870903

**Published:** 2021-02-09

**Authors:** Lei Gu, Qinghua Wu, Yin Yi, Zhengwen Yu

**Affiliations:** a School of Life Science, Guizhou Normal University, Guiyang, China; b Guangxi Botanical Garden of Medicinal Plants, Nanning, China

**Keywords:** Lonicera hypoglauca, Caprifoliaceae, complete chloroplast genome, phylogenetic

## Abstract

*Lonicera hypoglauca* Miq, which is widely distribute in south China, is an important Chinese plant used in traditional medicine. Here we report the first complete chloroplast (cp) genome sequence of this species. The circular cp genome is 154,581 bp in size, including a large single-copy (LSC) region of 88,379 bp and a small single-copy (SSC) region of 18,646 bp, which were separated by two inverted repeat (IR) regions (IRA and B, 23,778 bp each). A total of 121 genes were annotated, including 8 ribosomal RNAs (rRNAs), 33 transfer RNAs (tRNAs) and 80 protein-coding genes (PCGs). Phylogenetic analysis of 20 representative members within the Caprifoliaceae showed that *L. hypoglauca* is closely related to the *Lonicera macranthoides*. This study provides important genetic information for future systematic and evolutionary studies of *L. hypoglauca*.

*Lonicera hypoglauca* Miq, which is classified in the Caprifoliaceae, is widely distributed in southern China and Japan. The flower bud (rich in chlorogenic acid) of this plant is used as an herbal medicine and has been prescribed to treat various infectious diseases, such as fever, febrile blood dysentery and throat numbness (Zhang et al. 2008; Li et al. 2015). Pheophytin a, which can be extracted from the *L. hypoglauca* leaves, is an effective inhibitor of chronic hepatitis C virus NS3 protease (Wang et al. 2009). Some compounds (Bisflavonoid and loniceraflavone) in the leaves of *L. hypoglauca* significantly inhibit the activity of xanthine oxidase (Chien et al. 2009). Although the medicinal effect of *L. hypoglauca* have been fully studied, the complete chloroplast (cp) genome of *L. hypoglauca* has not been reported. In this research, we assembled and determined the cp genome sequence of *L. hypoglauca* as a resource for future study of this medicinal plant.

Young and healthy leaf samples were collected from Guangxi Botanical Garden of Medicinal Plants, Nanning, Guangxi Zhuang Autonomous Region, China (22°51′17.77″N, 108°24′48.15″E, 95 m above sea level). The leaf specimen (accession number: GZNUYZW202002001) was deposited in the herbarium of School of Life Sciences, Guizhou Normal University (Jinghua Mu, 842422147@qq.com). The total genomic DNA (No. YZW202002002) was extracted using the DNAsecure Plant Kit (TIANGEN, Beijing) and stored at −80 °C in the laboratory (room number: 1403) of School of Life Science, Guizhou Normal University. A total concentration of 800 ng of DNA served as the input material for the DNA sample preparations. Sequencing libraries were generated using NEB Next^®^ Ultra DNA Library Prep Kit for Illumina^®^ (NEB, USA). The library preparations were sequenced on an Illumina platform and 150 bp paired-end reads were generated. The filtered reads were assembled using the program GetOrganelle (Jin et al. 2019) with *Lonicera macranthoides* (GenBank accession number: MH579750) as the reference genome. The assembled cp genome was annotated using the online software GeSeq (Tillich et al. 2017). The complete chloroplast genome sequence was submitted to GenBank under the accession number MW186761.

The length of the complete cp genome sequence of *L. hypoglauca* is 154,581 bp, consisting of a large single-copy (LSC, 88,379 bp) region, a small single-copy (SSC, 18,646 bp) region and two inverted repeats (IRA and IRB) regions of 23,778 bp each. In total, 121 genes were predicted, including 80 protein coding (PCGs), 8 rRNA and 33 tRNA genes. Among the assembled genes, 4 rRNAs (*rrn16*, *rrn23*, *rrn4.5* and *rrn5*), 2 PCGs (*rps7* and *ndhB*) and 7 tRNAs (*trnA-UGC*, *trnH-GUG*, *trnI-GAU*, *trnL-CAA*, *trnN-GUU*, *trnR-ACG* and *trnV-GAC*) occurred as double copies. One PCG (*rps12*) occurred in three copies. Intron-exon analysis showed the majority (102 genes, 84%) of the genes do not display introns, whereas 19 (16%) genes contained introns.

To further understand the phylogenetic history of *L. hypoglauca*, 20 cp genome sequences of the Caprifoliaceae (12 *Lonicera* species, 1 *Heptacodium* species, 1 *Triosteum* specie, 2 *Dipelta* species, 1 *Weigela* species and 3 *Patrinia* species) were downloaded from GenBank to construct the phylogenetic trees through maximum-likelihood (ML) analysis. The ML tree was performed using RAxML (Version 8.0.19, Model: GTRGAMMA) (Stamatakis 2014) with 1,000 bootstrap replicates. The phylogenetic tree indicated that *L. hypoglauca* was fully resolved in a clade with *L. macranthoides*, sister to two other species of *Lonicera*, *L. confusa* and *L. japonica* (Figure 1). Compared to other Flos Lonicerae members, *L. hypoglauca* was also fully resolved in a clade with *L. macranthoides* according to *rbcL* gene sequence analysis (Li et al. 2012). Because of the closely evolutionary relationship between *L. macranthoides* and *L. hypoglauca*, in comparison with other DNA barcodes, only the *psbA-trnH* intergenic spacer sequence had appropriate mutation sites to distinguish *L. macranthoides* and *L. hypoglauca* (Sun et al. 2011).

**Figure 1.**
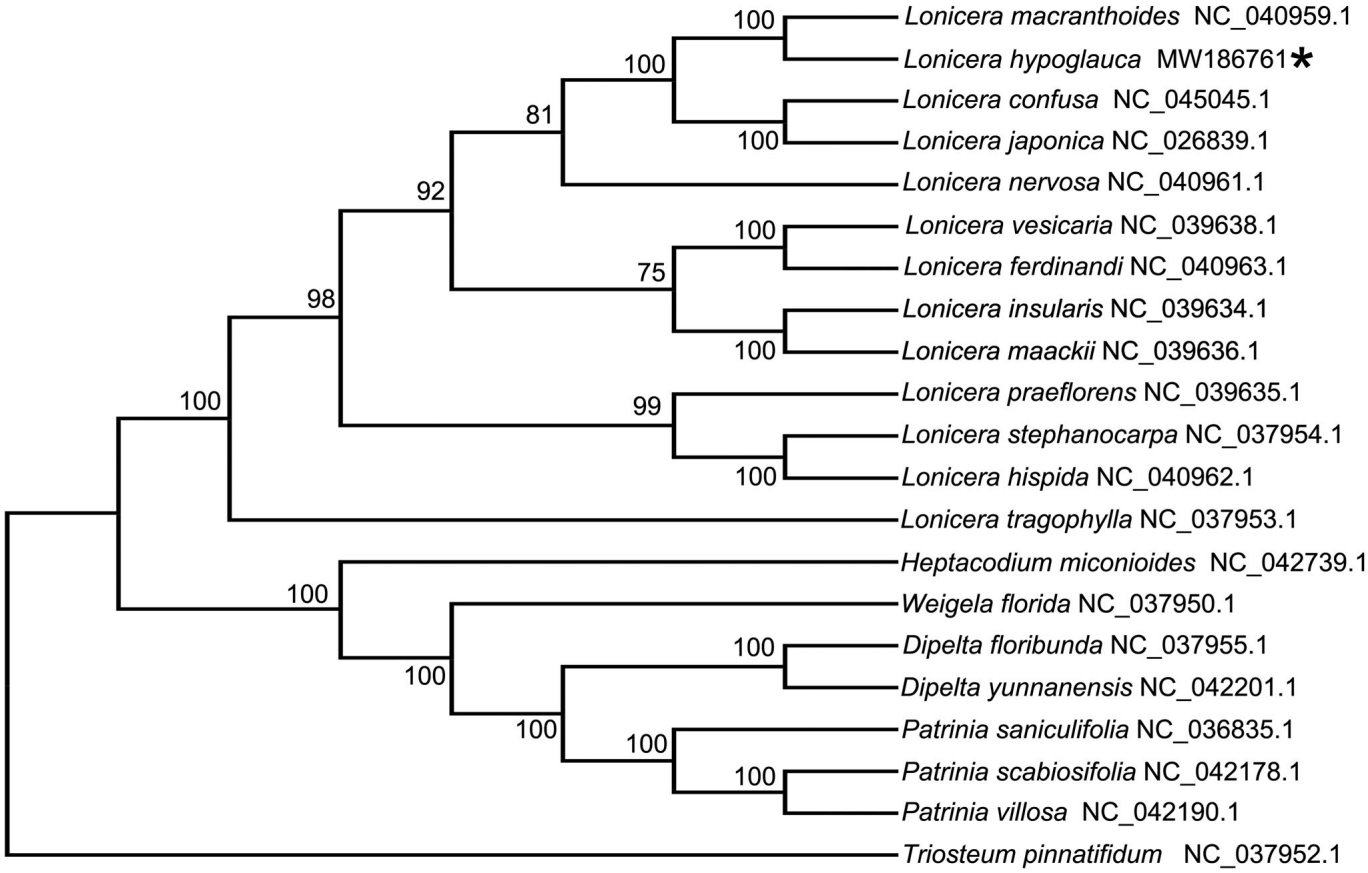
Maximum likelihood tree based on the complete cp genome sequences of 20 species from the Caprifoliaceae. GenBank accession numbers follow the binomials included in the figure. Shown next to the nodes are bootstrap support values based on 1,000 replicates.

## Data Availability

The complete chloroplast genome data that support the findings of this study are openly available in GenBank of NCBI at [https://www.ncbi.nlm.nih.gov] (https://www.ncbi.nlm.nih.gov/) under the accession number MW186761. The associated BioProject, SRA, and Bio-Sample numbers are PRJNA674956, SRX9460983, and SAMN16684231, respectively.
